# COVID-19 vaccine acceptance and rejection in an adult population in Bosnia and Herzegovina

**DOI:** 10.1371/journal.pone.0264754

**Published:** 2022-02-28

**Authors:** Adnan Fojnica, Ahmed Osmanovic, Nermin Đuzic, Armin Fejzic, Ensar Mekic, Zehra Gromilic, Imer Muhovic, Amina Kurtovic-Kozaric

**Affiliations:** 1 Institute of Biochemistry, Graz University of Technology, Graz, Austria; 2 Olawell Inc., Manchester, Massachusetts, United States of America; 3 International Burch University, Sarajevo, Bosnia and Herzegovina; 4 Department of Molecular biology, University of Vienna, Vienna, Austria; 5 Department of Pathology, Cytology and Human Genetics, University Clinical Center Sarajevo, Sarajevo, Bosnia and Herzegovina; Institute for Advanced Sustainability Studies, GERMANY

## Abstract

**Background:**

Bosnia and Herzegovina is among ten countries in the world with the highest mortality rate due to COVID-19. Lack of lockdown, open borders, high mortality rate, no vaccination plan, and strong domestic anti-vaccination movement present serious COVID-19 concerns in Bosnia and Herzegovina. In such circumstances, we set out to study 1) the willingness of general public to receive the vaccine, 2) factors that affect vaccine rejection, and 3) motivation for vaccine acceptance.

**Methods:**

A cross-sectional study was conducted among 10471 adults in Bosnia and Herzegovina to assess the acceptance or rejection of participants toward COVID-19 vaccination. Using a logistic regression model, we examined the associations of sociodemographic characteristics with vaccine rejection, reasons for vaccine hesitancy, preferred vaccine manufacturer, and information sources.

**Results:**

Surprisingly, only 25.7% of respondents indicated they would like to get a COVID-19 vaccine, while 74.3% of respondents were either hesitant or completely rejected vaccination. The vaccine acceptance increased with increasing age, education, and income level. Major motivation of pro-vaccination behavior was intention to achieve collective immunity (30.1%), while the leading incentive for vaccine refusal was deficiency of clinical data (30.2%). The Pfizer-BioNTech vaccine is shown to be eightfold more preferred vaccine compared to the other manufacturers. For the first time in Bosnia, vaccine acceptance among health care professionals has been reported, where only 39.4% of healthcare professionals expressed willingness to get vaccinated.

**Conclusion:**

With the high share of the population unwilling to vaccinate, governmental impotence in securing the vaccines supplies, combined with the lack of any lockdown measures suggests that Bosnia and Herzegovina is unlikely to put COVID-19 pandemic under control in near future.

## Introduction

**Fig 1:**
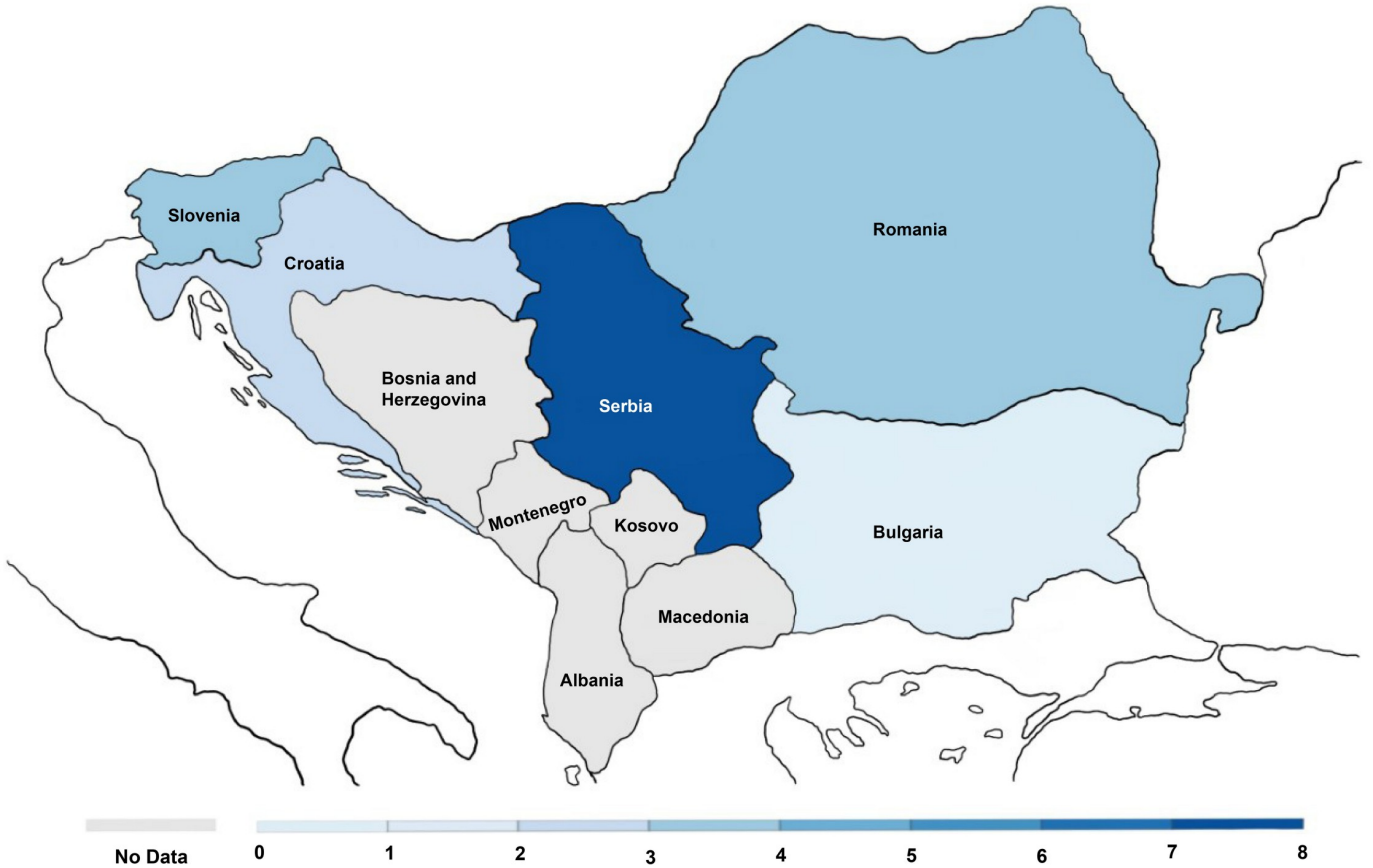
COVID-19 vaccine doses administered per 100 people, as of March 02, 2021 in the Western Balkans. Retrieved from Our World in Data on March 02, 2021. The map is created in Sketchbook.

On 1st March 2020, the World Health Organization (WHO) characterized the coronavirus disease 2019 (COVID-19) as a pandemic [1]. Since the first registered case of COVID-19 in December 2019 until March 2021, there were more than 100 million officially registered cases of COVID-19 and more than 2 million persons have passed away due to COVID-19 [1, 2]. Consequently, the rapid development of a COVID-19 vaccine was a global imperative [3]. In 2021, there are currently a few vaccines that passed the third phase of clinical trial and they are being distributed all over the world [4]. As of July 2021, the first dose was administered to 51% of population in high income nations, as compared to 1% of low income countries [5, 6] In the US and EU, Pfizer-BioNTech and Moderna vaccines have been approved [7], while European Medicines Agency (EMA) has recommended the approval of the AstraZeneca COVID-19 vaccine [8]. Safety and efficiency of the COVID-19 vaccine has been also confirmed for the Sputnik V [9]. Additionally, the National Medical Products Administration in China has given approval for the COVID-19 vaccine made by Sinovac Biotech [10]. Regarding the Balkans, vaccination has not started in Albania, Bosnia and Herzegovina (B&H), Kosovo, Montenegro, and North Macedonia (Fig 1). In B&H, the media have reported that in Republic of Srpska, an entity of B&H, around 2000 doses of Sputnik V COVID-19 vaccine have been distributed among healthcare workers [11].

In B&H, ~120000 cases have been officially registered until February 2021 (3.4% of the whole population) and almost 5000 deaths (4.16% of all cases). The peak of infection (2^nd^ wave) was from 31 October to 13 November 2020 [12]. Currently, in B&H there are around 400 active cases, with ~93 new confirmed cases daily per million people and ~4 deaths daily per million people [12]. In January 2021, B&H was 4th among the countries with the highest mortality rate due to COVID-19 with 123 deaths reported per 100000 [13]. Even though some COVID-19 measures are present such as the curfew from 23:00 to 5:00 h and the ban of public gatherings for >50 persons indoors and 100 persons outdoors, they are not enforced. There is no lock-down, borders are open, schools and universities are partially opened, while shopping centers, restaurants, ski centers, and bars are working as usual. Above all, a trend in vaccine rejection seen over the years, crisis with the ongoing pandemic, fast development of COVID-19 vaccines, and strong opposition by anti-vaccination movement led to the lack of COVID-19 vaccine acceptance on global scale as well as in B&H [14–20]. Understanding the vaccine hesitancy and reasons behind is vital for immunization programs, convincing vaccine opposition, and development of policies for more effective public health education [21–24].

The aim of this study was to collect and analyze data on the willingness of the public to be vaccinated and examine factors that affect vaccine rejection or acceptance. Additionally, we examined if vaccine rejection was affected by education, income, profession, and age.

## Materials and methods

### Research design, sampling, and ethics

We conducted a cross-sectional electronic survey study about COVID-19 vaccine acceptance in B&H from January 26^th^ to February 2^nd^, 2021. Answers were collected from a total of 10471 participants. Eligibility criteria included being age 18 or older and currently living in B&H. The survey was developed in the local language and created using Google’s online survey platform. The study was approved by the Ethics Committee of the Faculty of Engineering and Natural Sciences, International Burch University (04-51/21). Informed consent was obtained online when participants had the possibility to read basic information about the study before proceeding to answer the survey. All the study participants were informed that the data would be used only for research purposes and their individual answers would not be available to the public. According to Google’s privacy policy, all survey responses were anonymous and confidential. The survey was delivered to respondents via e-mails, research and employment-oriented online services (ResearchGate^™^ and LinkedIn^™^), and other social media platforms such as Facebook^™^, Skype^™^, and Viber^™^).

### Variables

The participants responded to a total of 11 items. Independent variables are grouped into three categories: 1) socio-demographic information (5 questions), (2) acceptance of COVID-19 vaccines (3 questions) and (3) knowledge about COVID-19 vaccine (3 questions). Socio-demographic questions included gender, level of education, profession, age, and monthly income. Gender was categorized as male, female or other. The level of education was defined as elementary school, high school, undergraduate degree, and postgraduate degree (master or doctorate). The profession was classified into five categories including medical professionals, educational sector, business sector, catering and service industry, and others. The age was categorized into four different groups: 18–30, 31–50, 51–64, and 65 years or older. Monthly income was defined as 250 EUR or less, 250–450 EUR, and 450 EUR or more.

COVID-19 vaccines acceptance and knowledge were assessed in a range of vaccine-related questions. Respondents were asked to claim whether they will choose to vaccinate or not (this item was recoded into dichotomous vaccine acceptance variable, with levels: ‘Yes’ and ’No / Only if I will have to / Maybe later‘). Additionally, participants were asked to corroborate their choice with rationale for or against vaccination having the ability to select multiple options. Furthermore, participants were asked to state their major source of information about health implications of COVID-19 vaccines. The respondents willing to be vaccinated were asked to indicate which vaccine manufacturer(s) would be their personal choice: Pfizer—BioNTech (Germany), Oxford-AstraZeneca (United Kingdom), Modern (USA), Sputnik V (Russia), or Sinovac (China), and to choose one or more reasons for the choice.

### Statistical analysis

The survey entries were converted to CSV format and prepared in Microsoft Excel. Data quality checks were ensured before the analysis using five main criteria: accuracy, relevancy, completeness, timeliness, and consistency. Statistical analysis included computing descriptive statistics of the data regarding the frequencies and percentages calculated for each category of the demographic set of questions. Binary logistic regression was employed to examine how independent demographic variables (gender, level of education, profession, age, and monthly income) affected dichotomous variable vaccine acceptance. Each nominal independent variable was recoded into binary, so-called, dummy variables. The assumptions for performing binary logistic regression were not violated. The dichotomous vaccine acceptance was used as the outcome. Odds ratio (OR) with a 95% confidence interval (CI) was used to assess the strength of association between each independent variable and the outcome. All analyses were performed using the R programming language (R Foundation for Statistical Computing, Vienna, Austria. http://www.R-project.org/).

## Results

Table 1 summarizes the set of demographic data including age, gender, education, monthly income, and profession. Women were 52.3% respondents of the survey and 53.9% were between 18 and 30 years old. More than half of the participants (53.1%) had monthly income of 450 EUR or more (average salary is about 450 EUR). About half of the respondents (51.9%) had a university degree. Significant number of healthcare professionals (15%) took part in our study.

**Table 1:** Summary of participants’ demographic data.

Variables	Overall
	n (%)
**Overall**	**10471 (100)**
**Gender**	
Male	4965 (47.4)
Female	5476 (52.3)
Others	30 (0.3)
**Level of education**	
Elementary school	159 (1.5)
High school	4878 (46.6)
Undergraduate degree	3757 (35.9)
Postgraduate degree (Master or Doctoral degree)	1677 (16)
**Profession**	
Medical professionals	1570 (15)
Education sector	936 (8.9)
Business sector	1639 (15.7)
Catering and service industry	721 (6.9)
Others	5605 (53.5)
**Age group in years**	
18–30	5649 (53.9)
31–50	4210 (40.2)
51–64	544 (5.2)
65+	68 (0.6)
**Total monthly income**	
250 EUR or less	2522 (24.1)
250–450 EUR	2384 (22.8)
900 EUR or more	5565 (53.1)

**Fig 2:**
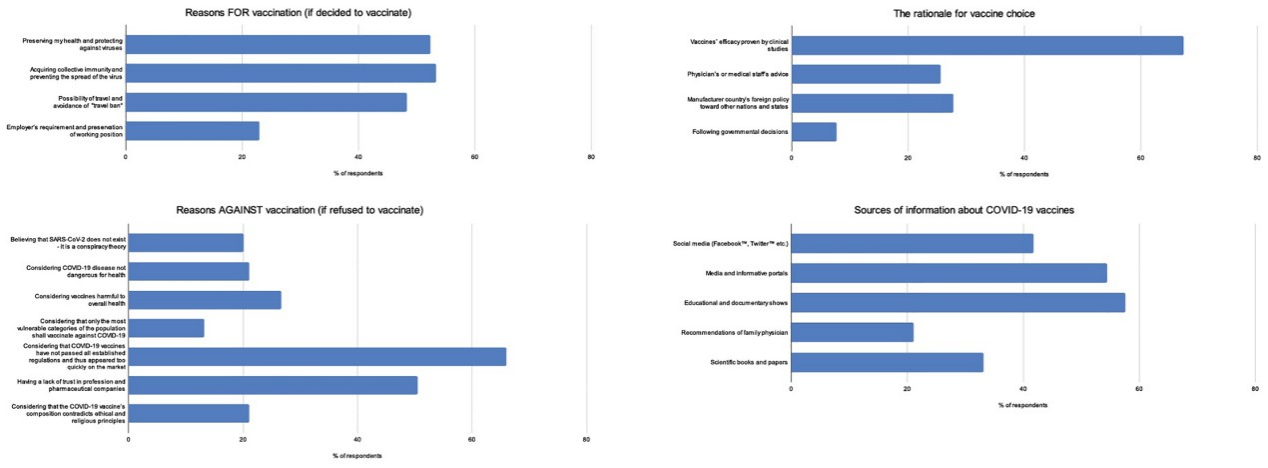
Reasons for vaccine acceptance, rejection, sources of information, and rational for vaccine choice.

Overall, 25.7% (2695 of 10461) of respondents indicated they are willing to get a COVID-19 vaccine, while 74.3% of respondents hesitated to get vaccinated (37.4% would not vaccinate, 13.7% respondents would vaccinate only if obliged, and 23.2% will wait for additional clinical studies to decide). Main reasons for vaccine acceptance, rejection, sources of information, and rational for vaccine choice are given Fig 2. Detailed breakdown of vaccine questions is available in S1 Table. We treated the three answers: ’No’, ’Only if I will have to’ and ’Maybe later’ as one group because they show similar trends in their answers.

Table 2 summarizes 5 binary logistic regression regarding vaccine acceptability against demographics (age, gender, monthly income, education, and profession). Accordingly, age, education, occupation and income significantly affected vaccine acceptance (p < .05), while sex of the participant did not (p > .05). People aged 31–50, 51–64 and 65+ were more likely to accept the vaccine than those who were aged 18–30. This difference was strongest (odds ratio (OR) = 4.61; 95% confidence interval (CI) (2.74, 7.77)) when respondents aged 65+ were compared to the youngest age cohort. The logistic regression suggests no significant distinction in the response to vaccine acceptance based on the gender.

**Table 2:** Beta coefficients and odds ratios of predictors of COVID-19 vaccine acceptance when comparing the answers ’Yes’ with ’No / Only if I will have to / Maybe later’.

Variable	Level comparison	Beta coefficients (95% CIs)	Odds ratios (95% CIs)
**Age**	31–50 vs 18–30	0.52 (0.42, 0.62)***	1.69 (1.53, 1.87)***
	51–64 vs 18–30	1.10 (1.00, 1.30)***	3.15 (2.59, 3.82)***
	65 or more vs 18–30	1.50 (1.00, 2.10)***	4.61 (2.74, 7.77)***
**Sex**	Female vs male	-0.01 (-0.10, 0.09)	0.99 (0.90, 1.10)
	Others vs male	-0.71 (-1.70, 0.18)	0.49 (0.17, 1.20)
**Education**	High school vs primary school	0.68 (0.18, 1.20)*	1.97 (1.20, 3.44)*
	Graduate vs primary school	1.50 (1.00, 2.10)***	4.57 (2.78, 8.01)***
	Postgraduate vs primary school	1.70 (1.10, 2.20)***	5.21 (3.14, 9.18)***
**Occupation**	Business sector vs medical professionals	-0.59 (-0.75, -0.44)***	0.55 (0.47, 0.65)***
	Catering and Service industry vs medical professionals	-0.70 (-0.94, -0.47)***	0.50 (0.39, 0.63)***
	Educational sector vs medical professionals	-0.93 (-1.10, -0.74)***	0.40 (0.33, 0.48)***
	Others vs medical professionals	-0.62 (-0.75, -0.49)***	0.54 (0.47, 0.61)***
**Income**	250 EUR- 450 EUR vs 250 EUR or less	-0.31 (-0.45, -0.16)***	0.74 (0.63, 0.85)***
	450 EUR or more vs 250 EUR or less	0.17 (0.04, 0.29)**	1.18 (1.04, 1.34)**

*, *p* < .05;

**, *p* < .01;

***, *p* < .001.

Higher income was positively associated with vaccine acceptance. People earning 450+ EUR per month were 1.18 (95 CI% (1.04, 1.34)) times more likely to respond positively to the vaccine acceptance question than people earning 250 EUR and less. Higher levels of education were also associated positively with vaccine acceptance. Respondents from the postgraduate group were 5.21 (95 CI% (3.14, 9.18)) times more likely to respond positively compared to participants having only primary school education. Medical health professionals were more likely to get vaccinated compared to other professions. In fact, educational sector had 60% lower odds of vaccine acceptance compared to the health professionals.

Major predictors behind vaccination were achieving collective immunity (30.11%) and concern regarding personal health (29.57%), following avoidance of “travel ban” (27.31%) and employer request (13.00%). The Pfizer-BioNTech would be chosen by 50.62% participants willing to vaccinate, while Sinovac vaccines would be preferred for only 6.44% of them. Effectiveness shown in clinical trials is the main motive for Pfizer’s vaccine choice. Most objections to vaccination are due to insufficient clinical trials (30.11%), 23.08% respondents perceive pharmaceutical companies as self-serving enterprises. Significant numbers recognize vaccines as harmful (12.23%), 9.63% participants identify COVID-19 disease as harmless to their health, while an identical portion of respondents reject vaccines due to religious motives. For 9.19% participants SARS-CoV-2 virus is just a conspiracy theory, while 6.05% individuals assessed vaccines as necessary only for clinically vulnerable citizens.

## Discussion

To the best of our knowledge, we report the lowest COVID-19 vaccine acceptance in the world, where only 25.7% participants demonstrated willingness to receive vaccination against SARS-CoV-2; the lowest COVID-19 vaccination acceptance levels reported previously were in Poland (37%), Slovakia (41%), Romania (44%) and Czech Republic (49%) [24, 25]. Understanding of vaccination refusal and reasons for rejection among citizens in B&H is of great importance as reports from January 2021 list B&H as fourth in the world in terms of deaths per 100000 inhabitants, right after Slovenia, Belgium and San Marino [13]. Observed data should be used to raise awareness among the population and reach those strongly advocating against COVID-19 vaccination programs.

Logistic regression outputs for vaccine acceptability demonstrate important discrepancies across diverse categories in the survey. Participants with above average income were more likely to accept vaccination compared to those having minimum wage. Findings suggest participants with primary school education were more prone to reject vaccination compared to participants having higher levels of education. Observed data are in accordance with studies previously conducted [24].

Our study suggests no significant distinction in the response to vaccine acceptance based on the gender. However, we see a trend where women seem to be more hesitant regarding COVID-19 vaccines, while men are slightly more prone to vaccination, diverting from the trend of higher medical care service utilization among women [26]. Additionally, we observed age-related associations with vaccine acceptance. Older people were more likely to report that they would take a vaccine, whereas respondents aged 18–30 years had the highest rate of vaccination refusal [25, 27, 28]. We also observed the effect of education on vaccine acceptance, where more educated population was more likely to accept vaccination, as observed in previously published data [29–34]. The main reasons could be a better understanding and trust of the science and scientific methods.

In this study, COVID-19 vaccine acceptance among health care professionals has been examined and compared to the other professions. Only 39.4% of healthcare professionals are willing to accept COVID-19 vaccination, while others are hesitant or strongly refusing vaccination. This confirms concerns raised by Arapovic et al. in 2019, regarding lower measles vaccine acceptance among medical professionals in B&H, as they directly communicate with patients and shape their perspective toward vaccination [35, 36].

Major drivers of pro-vaccination behavior were the intention to achieve collective immunity and personal protection. The participants prefer Pfizer-BioNTech vaccines up to eightfold more compared to the other vaccine manufacturers; the major reasons for choosing Pfizer-BioNTech vaccine were high effectiveness in clinical trials and manufacturer’s country of origin [37–41]. Rationale for vaccine choice showed that the lowest percent of respondents (7.7%) would follow government guidelines. Thus, the confidence in the government is low, as the population witnesses various political and socio-economic crises in the post-war period.

Strong domestic anti-vaccination movement has started several years ago against common pediatric vaccines such as measles [14, 15]. Anti-vaccination groups target local media and online platforms to spread misleading health information and address controversial arguments such as the economic benefit for pharmaceutical companies and tragic personal stories [16]. In our survey, media platforms and social networks were the main sources of information during the pandemic, which makes the high COVID-19 vaccine rejection understandable [16]. As the second major motivation for vaccine rejection, participants listed mistrust in pharmaceutical companies, followed by the assessment that the vaccines are harmful. Based on these results, scientific community and health care professionals advocating vaccines in B&H ought to be more presented to raise awareness and reach citizens looking for reliable information.

This study has several limitations, which we address here. Even though this study has a large number of participants, cross-sectional study design captures relevant information in a single defined point in time. This survey was conducted in February 2021, before the availability of COVID-19 vaccines and as such represents the attitude towards vaccination and may evolve over time as more data is available to the general population. The survey was conducted over the Internet, which may have inherent bias towards a more educated population. Data in this survey have been collected using online social networks, which often excludes citizens in the category of age 65 and older [42]. Only 0.6% of participants were >65 years. Since they represent a high-risk group and are more likely to accept vaccination, the acceptance rate may be larger than presented [24, 25]. However, the range of questions and the large population size could ameliorate this limitation, and provide a reasonable population model in assessing willingness for vaccine acceptance in B&H.

Besides demographic data, our study includes independent variables such as acceptance and knowledge about COVID-19 vaccines, but does not include variables such as mitigating factors: usage of face masks indoors, physical distancing, current health status, and prior positivity for SARS-CoV-2 [43]. For example, one of the possible limitations is that the survey did not have a question on prior positivity for SARS-CoV-2, which could change the participant’s attitude towards vaccination. In summary, additional research with the focus on the elderly category and mitigating factors would be beneficial to completely address these limitations. All raw data from this study is available to the public for further analyses.

According to current studies, herd immunity benefits are achievable if >70% of the population is vaccinated [44]. With the high share of the population unwilling to vaccinate, governmental impotence in securing the vaccines supplies, combined with the number of people unable to receive the COVID-19 vaccine (e.g., allergies), herd immunity is out of reach for the B&H population in the near future. In order to increase awareness regarding health benefits of vaccination and the historical role immunization had in eradication of many deadly diseases, people must be reached through main informing sources—educational programs and media. Additional efforts must be made to organize scientific panels and conferences for healthcare workers and physicians, as only 39.4% of them are willing to accept vaccination. Ideally, frontline medical professionals should make strong recommendations for vaccination, as well as share their personal experiences with COVID-19 vaccines. Finally, preparation for public acceptance of a COVID-19 vaccine must be carefully conducted before a vaccine becomes widely available. Based on this study, we urge the Bosnian government to develop strategies and COVID-19 vaccination implementation plans that would encourage citizens to accept a vaccination [45].

## Conclusion

With the high share of the population unwilling to vaccinate, governmental impotence in securing the vaccines supplies, combined with the lack of any lockdown measures suggests that Bosnia and Herzegovina is unlikely to put COVID-19 pandemic under control in near future.

## Supporting information

S1 Table Detailed breakdown of vaccine questions used in the survey. (DOCX)
