# Supplementary material for: COVID-19 vaccine acceptance and rejection in an adult population in Bosnia and Herzegovina
Source: PLoS One. 2022 Feb 28;17(2):e0264754. doi: 10.1371/journal.pone.0264754 (PMC8884480; doi:10.1371/journal.pone.0264754)
Supplement: S1 Table — (DOCX) [file pone.0264754.s001.docx]

**Supplementary Table 1.** Breakdown of COVID-19 vaccine questions

| **Variables** | **Overall** |
| --- | --- |
|  | **n (%)** |
| **Overall** | **10471 (100)** |
| Would you vaccinate against COVID-19? | |
| Yes | 2695 (25.7) |
| No | 3916 (37.4) |
| Only if obligatory (i.e. employer requires it) | 1433 (13.7) |
| Waiting for additional clinical studies to decide | 2427 (23.2) |
| Reasons FOR vaccination (if decided to vaccinate)  (more than one choice per response possible) | |
| Preserving my health and protecting against viruses | 3230 (52.3) |
| Acquiring collective immunity and preventing the spread of the virus | 3289 (53.3) |
| Possibility of travel and avoidance of "travel ban" | 2983 (48.3) |
| Employer’s requirement and preservation of working position | 1420 (23) |
| Reasons AGAINST vaccination (if refused to vaccinate)  (more than one choice per response possible) | |
| Believing that SARS-CoV-2 does not exist - it is a conspiracy theory | 1455 (20.1) |
| Considering COVID19 disease not dangerous for health | 1524 (21.1) |
| Considering vaccines harmful to overall health | 1936 (26.7) |
| Considering that only the most vulnerable categories of the population shall vaccinate against COVID19 | 957 (13.2) |
| Considering that COVID19 vaccines have not passed all established regulations and thus appeared too quickly on the market | 4778 (66) |
| Having a lack of trust in profession and pharmaceutical companies | 3653 (50.5) |
| Considering that the COVID19 vaccine’s composition contradicts ethical and religious principles | 1524 (21.1) |
| Sources of information used to learn about the health implications  of COVID19 vaccines (more than one choice per response possible) | |
| Social media (Facebook™, Twitter™ etc.) | 4375 (41.8) |
| Media and informative portals | 5694 (54.4) |
| Educational and documentary shows | 6031 (57.6) |
| Recommendations of family physician | 2207 (21.1) |
| Scientific books and papers | 3462 (33.1) |
| Which COVID19 vaccine will be your choice? (if decided to vaccinate)  (more than one choice per response possible) | |
| Pfizer-BioNTech (Germany) | 5833 (80.5) |
| Oxford-AstraZeneca (UK) | 1862 (25.7) |
| Moderna (USA) | 1763 (24.3) |
| Sputnik V (Russia) | 1323 (18.3) |
| Sinovac (China) | 742 (10.2) |
| The rationale for vaccine choice (more than one choice  per response possible) | |
| Vaccines’ efficacy proven by clinical studies | 4647 (67.3) |
| Physician’s or medical staff’s advice | 1767 (25.6) |
| Manufacturer country’s foreign policy toward other nations and states | 1924 (27.8) |
| Following governmental decisions | 532 (7.7) |
